# Antimicrobial Peptides: Diversity, Mechanism of Action and Strategies to Improve the Activity and Biocompatibility In Vivo

**DOI:** 10.3390/biom8010004

**Published:** 2018-01-19

**Authors:** Prashant Kumar, Jayachandran N. Kizhakkedathu, Suzana K. Straus

**Affiliations:** 1 Department of Chemistry, University of British Columbia, 2036 Main Mall, Vancouver, BC V6T 1Z1, Canada; pkumar02@chem.ubc.ca; 2 Centre for Blood Research, Department of Pathology and Laboratory Medicine, University of British Columbia, 2350 Health Sciences Mall, Life Sciences Centre, Vancouver, BC V6T 1Z3, Canada

**Keywords:** antimicrobial peptides, mechanism of action, chemical modification, delivery systems, bioconjugation, biocompatibility, proteolysis

## Abstract

Antibiotic resistance is projected as one of the greatest threats to human health in the future and hence alternatives are being explored to combat resistance. Antimicrobial peptides (AMPs) have shown great promise, because use of AMPs leads bacteria to develop no or low resistance. In this review, we discuss the diversity, history and the various mechanisms of action of AMPs. Although many AMPs have reached clinical trials, to date not many have been approved by the US Food and Drug Administration (FDA) due to issues with toxicity, protease cleavage and short half-life. Some of the recent strategies developed to improve the activity and biocompatibility of AMPs, such as chemical modifications and the use of delivery systems, are also reviewed in this article.

## 1. Antimicrobial Peptides: History and Diversity

**Figure 1:**
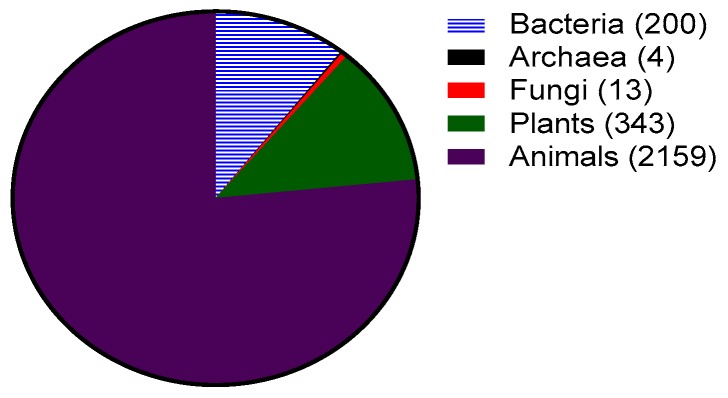
Sources of antimicrobial peptides (total 2818) as of September 2017 from the antimicrobial peptide database. Numbers obtained from http://aps.unmc.edu/AP/, accessed on 20 September 2017.

Antimicrobial peptides (AMPs), more recently known as host defense peptides, are found in virtually all forms of life. Antimicrobial peptides are produced by all organisms ranging from bacteria to plants, vertebrates and invertebrates (Figure 1). In bacteria, the AMPs benefit individual species by killing other bacterial species that may compete for nutrients and the same environmental niche. Bacterial AMPs known as bacteriocins are classified into two categories: lantibiotics and non-lantibiotics. Lantibiotics are AMPs containing the non-natural amino acid lanthionine. Nisin, a lantibiotic, was one of the first AMPs isolated and characterized from *Lactococcus lactis* in 1947 [1]. It is active against a variety of Gram-positive bacteria with a minimum inhibitory concentration (MIC) in the nanomolar range and has been used as a food preservative for 50 years with no significant development of resistance [2]. Other bacteriocins such as mersacidin have also been studied for their possible use against antibiotic-resistant Gram-positive bacteria [3].

Most AMPs reported to date are from eukaryotic origins such as plants, animals, and fungi (Figure 1). Since 1885, fluids such as blood, sweat, saliva, plasma, white blood cell secretions and granule extracts have been prized for their antimicrobial properties [4]. However, it was not till 1981 that Hans Boman reported that the hemolymph (plasma and blood) of silk moth (*Hyalophora cecropia*) contained AMPs known as cecropins [5]. These peptides are cationic, amphipathic and have broad spectrum activity (i.e., are active against multiple types of microorganisms such as Gram-positive and Gram-negative bacteria and fungi). The field grew further when Rober Leher, Shunji Natori and Michael Zasloff isolated and described defensins [6] (from mammalian macrophages), sacrotoxins [7] (from fly larvae) and magainins [8] (from the skin of frogs *Xenopus laevis*), respectively.

In eukaryotes, AMPs play an important role in innate immunity. Plants lack adaptive immunity (i.e., B cell and T cell mediated immunity) and hence AMPs play a fundamental role in their protection against infection by bacteria and fungi. The presence of genes encoding for plant AMPs can be found in a variety of plant species. Interestingly, all plant AMPs are cysteine rich and contain many disulfide bonds [9]. The best studied groups of plant AMPs include the thionins [10], plant defensins [11] and cyclotides [12]. Plant AMPs can be found in leaves, flowers, seeds and tubers [2].

Similar to plants, invertebrates lack an adaptive immune system and hence are completely dependent on the innate immune system for protection against infection. AMPs have been found in all invertebrates examined to date, which mostly includes insects and marine invertebrates [13]. AMPs can be found in the hemolymph, hemocytes (blood cells), phagocytes (white blood cells) and epithelial cells of these creatures [2]. As mentioned earlier, the first AMP (cecropins) from eukaryotes was discovered in silk moth, but cecropins are also found in fruit flies (*Drosophila).* Many other marine invertebrates such as shrimp, oysters and horseshoe crabs express AMPs [14,15] constitutively (i.e., via a gene that is transcribed and translated continually to make a protein or peptides). Tachyplesin and polyphemusin are two potent AMPs produced by horseshoe crabs which possess antibacterial and antifungal activity at low micromolar range [16]. Interestingly, as with some other AMPs, polyphemusin also shows antiviral activity against human immunodeficiency virus (HIV) [17].

Vertebrate immunity consists of both an innate and adaptive immune system. AMPs have been isolated and characterized from a variety of vertebrates such as fish, mammals and amphibians. Vertebrate AMPs can be isolated from a variety of cells, such as granules of white blood cells (phagocytes, neutrophils, macrophages, natural killer cells), epithelial tissue situated in the mouth, lungs, or skin, and bodily fluids [18,19,20]. Interestingly, amphibian skin glands have been a rich source of AMPs, with more than 500 AMPs reported to date [2]. Most vertebrate AMPs show direct antimicrobial activity at high concentration such as in the granules of white blood cells. However, some vertebrate AMPs have also been shown to perform critical functions in immune modulation and controlling inflammation [21,22,23,24,25,26]. The two most prominent groups of AMPs in vertebrates are cathelicidins and defensins, which will be discussed further in the next section. 

### 1.1. Categories of Antimicrobial Peptides

Antimicrobial peptides are a distinct and diverse class of molecules. With over 2800 peptides sequences reported to date, it is important to categorize AMPs. AMPs can be classified in many different ways, which can be based on structure, sequence, or mechanism of action. AMPs can also have a range of activities: from killing bacteria, to immune modulation or preventing biofilm formation, as well as anti-cancer (e.g., see Section 2.1 for more details) or anti-viral properties. As the activity of the peptides is dependent on the structure and the sequence, it is important to take both of these properties into account while grouping AMPs together. In this review, we will be focusing on eukaryotic cationic AMPs, which primarily act by killing bacteria (Section 2.1 and Section 2.2) or by modulating the immune system (Section 2.3). For recent reviews on anti-cancer or anti-viral properties of AMPs, the reader is invited to consult the references given [27,28].

**Figure 2:**
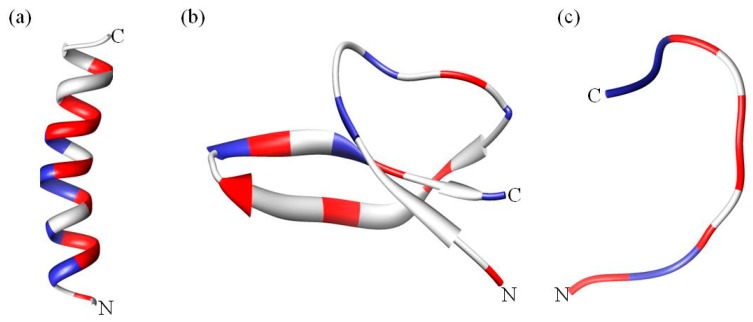
Structural diversity of antimicrobial peptides (AMPs). (**a**) the α helical magainin, (**b**) β sheet human defensin 5 and (**c**) extended coil indolicidin. Positively charged residues are colored blue whereas hydrophobic residues are red. The N- and C-termini are indicated. The figure was generated using CHIMERA software [57,58,59].

A classification based on structural features results in three broad subclasses, as listed in Table 1. The first subgroup contains AMPs that adopt an alpha helical structure and which are predominantly found in the extracellular matrix of frogs and insects. Most of these peptides are unstructured in aqueous solution but become structured when in contact with trifluoroethanol, detergents/surfactants above critical micellar concentration such as sodium dodecyl sulfate (SDS) [29], micelles and liposomes [30] (Figure 2a). An extensively studied human AMP in this subgroup is LL-37 (Table 1), which is a member of the cathelicidins. Cathelicidins are one of the most diverse AMPs of vertebrates, mainly found in mammals such as mice, sheep, goat, horses and bovines [31]. Cathelicidin AMPs range from 12–80 amino acids and can adopt a variety of other structures (Table 1). In addition to their antimicrobial activity, cathelicidins such as LL-37 play an important role in immunomodulatory and inflammation responses [23]. Another good example are the α helical magainins (Table 1), which were originally isolated from the African clawed frog *Xenopus laevis* and are active against Gram-positive and Gram-negative bacteria, fungi, yeast and viruses [32]. The structure and function relationship of the magainins has been well studied [33,34]. These AMPs were the first to be tested in the clinic, but ultimately failed [2]. However, the magainin analogue pexiganan is currently in clinical trials [35]. 

Finally, another example of alpha helical AMPs is the aurein family. Aurein peptides are secreted from the granular dorsal glands of the Australian Green and Golden Bell Frog *Litoria aurea* and the Southern Bell Frog *L. raniformis*. There are more than 30 aurein peptides from five different families, ranging from the short active aurein peptides (aurein 1–3) to the longer peptides such aurein 4.1 and 5.1 which are not active [36]. Most aurein peptides are active against Gram-positive bacteria such as *Staphylococcus aureus* and *Staphylococcus epidermidis*. Some peptides, such as aurein 1.2, 3.2 and 3.3, display their strongest activity against 30–50 different types of cancer [37]. The mechanism of action and structure of aurein 2.2 has been extensively studied in recent years [38,39,40]. Early studies on aurein 2.2 have shown that it is important to study the bilayer perturbation in membrane models made from phosphatidylcholine (PC) and phosphatidylglycerol (PG) rather than PC alone, indicating that electrostatic interactions are important in the lipid–peptide interaction [38]. Also, the truncation of the N-terminus in aurein 2.2 leads to the loss of antimicrobial activity but makes the peptide immunomodulatory [41]. In a more recent study Wenzel at al. showed that aurein 2.2 forms ion selective pores, permitting the translocation of ions such as potassium and magnesium. In addition aurein 2.2 causes membrane permeabilization, which disrupts the membrane potential and decreases the energy supply of the cells leading to cell death [42]. Another important feature of alpha helical peptides is that most require amidation at the C-terminus for higher antimicrobial activity (Table 1). The amidation of the C-terminus enhances the electrostatic interaction between the positively charged peptide and the negatively charged bacterial membrane. This interaction stabilizes the helical structure at the membrane interface [43].

The second subclass of AMPs predominantly adopts a β sheet structure (Figure 2b). This class includes AMPs such as protegrins (from the cathelicidin family), defensins and tachyplesins. Interestingly, nearly all β sheet AMPs contain cysteine residues that are conserved and form disulfide bonds. In defensins, the disulfide bonds provide structural stability and minimize protease degradation [44]. β sheet AMPs are more structured in solution and do not undergo major structural changes when going from an aqueous environment to a membrane environment [45]. Defensins are the largest group of AMPs that are further categorized in sub-families on the basis and location of the disulfide bond (Table 1). Defensins are also involved in antibacterial, antifungal, antiviral, immune and inflammation responses [23]. Knockout and transgenic mice experiments have indicated that α defensins are critical for protection against *Escherichia coli* [46] and *Salmonella enterica* [47]. Tachyplesins and polyphemusin (arginine rich, ~30% by sequence) are other β sheet AMPs that were isolated from hemocytes of horseshoe crabs [48,49].

The third and last subclass of AMPs has a unique extended coil structure. Most of the AMPs in this category are from the cathelicidin family and consist of two or more proline residues, which are known to break secondary structural elements such as α helices or β sheets [60]. Indolicidin is a tryptophan rich AMP isolated from bovine neutrophils and consists of only 13 amino acids [61]. Nuclear magnetic resonance (NMR) and circular dichroism (CD) studies reveal that indolicidin forms a unique “membrane-associated peptide structure” with well defined extended structure in the presence of micelles [61,62] (Figure 2c). In a more recent study, Falcao et al. [63] discovered that the C-terminal fragment of the crotalicidin peptide (crotalicidin 15–34) showed antibacterial activity against Gram-negative bacteria and antitumor activity. NMR studies of the active crotalicidin 15–34 revealed that the AMP mostly adopts an extended structure (83%) and was only 17% α helical. The N-terminus (crotalicidin 1–14) was fully α helical but inactive.

### 1.2. Common Properties of Antimicrobial Peptides

Although AMPs are a diverse group of molecules in terms of sequence, structure and sources, there are several properties that are common to almost all AMPs. Firstly, AMPs display a net positive charge ranging from +2 to +13 and may contain a specific cationic domain. The cationic nature can be attributed to the presence of lysine and arginine (and sometimes histidine) residues. Many studies have demonstrated the correlation between charge and antimicrobial activity of AMPs [64,65,66,67,68]. Increasing the charge of magainin 2 from +3 to +5 improved the antibacterial activity against both Gram-positive and Gram-negative bacteria, but an increase to +6 or +7 lead to increased hemolytic activity and loss of antimicrobial activity [65]. The loss of antimicrobial activity may be due to the fact that an extremely strong interaction between the peptide and the phospholipid head group would prevent translocation of the peptide into the inner leaflet of the membrane [69]. 

Secondly, hydrophobicity is a key feature for all AMPs and is defined as the percent of hydrophobic residues such as valine, leucine, isoleucine, alanine, methionine, phenylalanine, tyrosine and tryptophan in the peptide sequence (typically 50% for AMPs). Hydrophobicity governs the extent to which the water-soluble AMPs will be able to partition into the membrane lipid bilayer. It is required for membrane permeabilization; however, excessive levels of hydrophobicity can lead to mammalian cell toxicity and loss of antimicrobial selectivity [69,70,71]. Chen et al. examined the influence of hydrophobicity in a synthetic α helical AMP (V13KL) on the antimicrobial activity and hemolysis of human red blood cells (RBCs) [71]. The results suggest that there is an optimal hydrophobicity needed for good antimicrobial activity. Sequences with hydrophobicities below and very much above this threshold made the peptides inactive [71]. The decrease in activity when the hydrophobicity is high may be due to the increased likelihood of dimerization, thereby preventing access of the peptide to the bacterial membrane. Additionally, increasing the hydrophobicity of the non-polar face of the amphipathic α helix also enhances the lysis of RBCs. This may be due to the membrane discrimination mechanism as peptides with higher hydrophobicity penetrate deeper into the hydrophobic core of the RBC membrane [71].

The last feature shared by all antimicrobial peptides is amphipathicity. Amphipathicity refers to the relative abundance of hydrophilic and hydrophobic residues or domains within the AMPs. It can be thought of as the balance between the cationic and hydrophobic residues, not just at the primary sequence level, but also in terms of the 2D or 3D structure of the AMPs. Amphipathicity can be achieved by a number of peptide conformations such as the ones listed in Table 1, but the most elegant example is the α helix. The α helix allows the peptide to form two “faces”, namely the polar and nonpolar face referring to the arrangement of the hydrophobic and hydrophilic side chain of the residues in the helix (Figure 2a). Amphipathicity of AMPs can be reflected by calculating the hydrophobic moment which is the vector sum of individual amino acid hydrophobicity, standardized to an ideal helix [69] (calculated using e.g., http://www.bioinformatics.nl/emboss-explorer/). Interestingly, for α helical AMPs it was previously thought that disruption of the amphipathicity leads to an increase in antimicrobial activity and reduction in RBC lysis [56,72,73,74,75]; however, a recent study by Zhang et al. on melittin related peptides demonstrated that increased amphipathicity also leads to a decrease in RBC lysis [76]. Moreover, protegrin-1 and tachyplesin-1 are both β sheet AMPs. Protegrin-1 is more amphipathic than tachyplesin-1 and the increase in amphipathicity leads to a two-fold increase in hemolysis, but does not have a major effect on the antimicrobial activity, suggesting a link between amphipathicity and hemolysis [77]. On the other hand, analogues of indolicidin with increased charge and amphipathicity display a lower hemolytic activity while preserving the antimicrobial activity and hence increasing the therapeutic index of the peptides [78,79]. These studies suggest a complicated relationship between amphipathicity, hydrophobicity and net charge. It would seem rather that the different parameters play a unique role, depending on the peptide sequence. 

## 2. Mechanism of Antimicrobial Peptide Action

**Figure 3:**
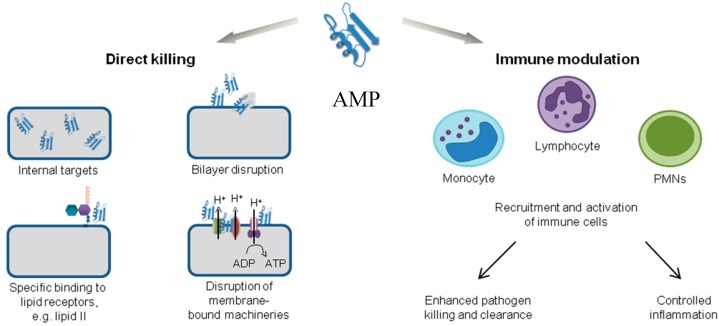
Various mechanisms of action of antimicrobial peptides. Adapted with permission from [54]. MN: polymorphonuclear neutrophils; ADP: adenoside diphosphate; ATP: adenoside triphosphate.

Antimicrobial peptides are unique molecules and their mechanism of action (MOA) has been studied extensively since they were discovered. It is important to understand the MOA of these AMPs to facilitate further development as therapeutic agents. It was originally thought that membrane targeting was the only MOA, but there is increasing evidence now that AMPs have other modes of action [54] (Figure 3). The MOA can be divided into two major classes: direct killing and immune modulation. The direct killing mechanism of action can be further divided into membrane targeting and non-membrane targeting, which will be the focal point of the following sections. 

### 2.1. Direct Killing: Membrane Permeabilizing Mechanism of Action 

The membrane targeting AMPs can have receptor mediated or non-receptor mediated interactions. The receptor mediated pathway mostly includes AMPs produced by bacteria and which are active in vitro in the nanomolar range, such as nisin [80]. The nisin peptide has two domains: the first domain binds with high affinity to the lipid II molecule, a membrane anchored cell wall precursor. The second region is the membrane-embedded pore-forming domain. Mesentericin is another example of a receptor mediated membrane targeting AMP [81].

**Figure 4:**
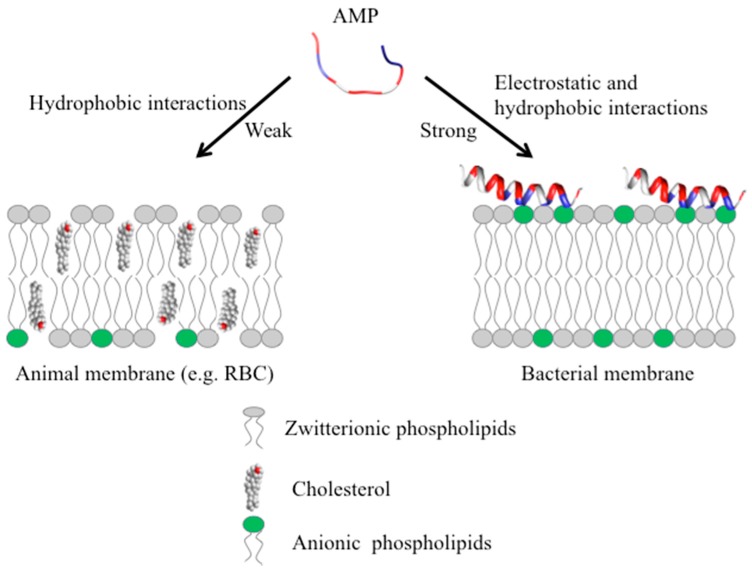
Initial interaction of cationic AMPs with the multicellular animal (**left**) or bacterial (**right**) membrane. RBC: red blood cell.

Most vertebrate and invertebrate AMPs target the membrane without specifically interacting with receptors. These AMPs are typically active in vitro against microbes at micromolar concentrations [82] and function by interacting with the components of the membrane. The outer surface of Gram-positive bacteria and Gram-negative bacteria contains teichoic and lipopolysaccharide, each conferring net negative charge on the surface allowing the initial electrostatic attraction with cationic AMPs [69,82]. More importantly, AMPs target a fundamental difference in design between the bacterial membrane and the membrane of multicellular animals. The outer monolayer (leaflet) (Figure 4) of the lipid bilayer in bacterial membranes is made up of mostly lipids with negatively charged head groups, such as PG and cardiolipin [83], whereas the outer leaflet of the animal membranes are made up of zwitterionic phospholipids such as PC, sphingomyelin and other neutral components such as cholesterol [84]. Most of the lipids with negatively charged head groups are in the inner leaflet facing the cytoplasm in animal membranes [84,85]. The positively charged AMP has strong electrostatic interactions with the negatively charged phospholipids on the outer leaflet of the bacterial membrane (Figure 4).

Interestingly, as mentioned above, some AMPs also display anticancer activity. In cancer cells, the asymmetry (Figure 4, left) between the inner and outer membrane in terms of the negatively charged phospholipids is lost, resulting in the increased abundance of negatively charged phosphatidylserine (PS) on the outer leaflet which enhances the interactions with AMPs [86]. Additionally, the over expression of other negatively charged biomolecules such as heparan sulfate, O-glycosylated mucins, sialylated gangliosides, and the increased transmembrane potential and membrane fluidity also allow AMPs to specifically target cancer cells [27]. Aurein 1.2 is highly active against approximately 50 different kinds of cancer cell lines and displays little toxicity [36,37]. 

Moreover, some AMPs are even sensitive to other properties of the lipids, i.e., not just the charge [87,88]. For instance, magainins can induce leakage more effectively in liposomes made of PG, an anionic phospholipid found predominantly in bacterial membranes compared to liposomes composed of negatively charged PS, a major phospholipid of animal membranes [87]. Lipids have different shapes depending on the relative size of the head group and hydrophobic tails. PS and PG have a molecular shape that is similar to a cone and cylinder, respectively, and hence display different membrane curvature properties [89]. On the other hand, the AMP polybia-MP1, causes more leakage in PS containing large unilamellar vesicles compared to PG (both anionic lipids) due to the lipid geometry and the synergy of the PS with other membrane components such as sphingomyelin/cholesterol domains [90]. The head group of PS contains both positive (amino) and negative (carboxylic) charges which allow specific peptide lipid interactions and hence higher activity compared to membrane containing PG [90]. The specific interactions are not only limited to anionic lipids as AMPs, such as plant cyclotides, can also bind specifically to zwitterionic lipid such as phosphatidylethanolamine (PE), which is abundant in the surface of bacterial membranes and also found in great quantity in the inner cytoplasmic leaflet of animal membranes [91,92]. These studies suggest that membrane charge is not the only factor that is important for the initial interaction, but other properties such as membrane curvature may also play an important role [87,93,94]. 

**Figure 5:**
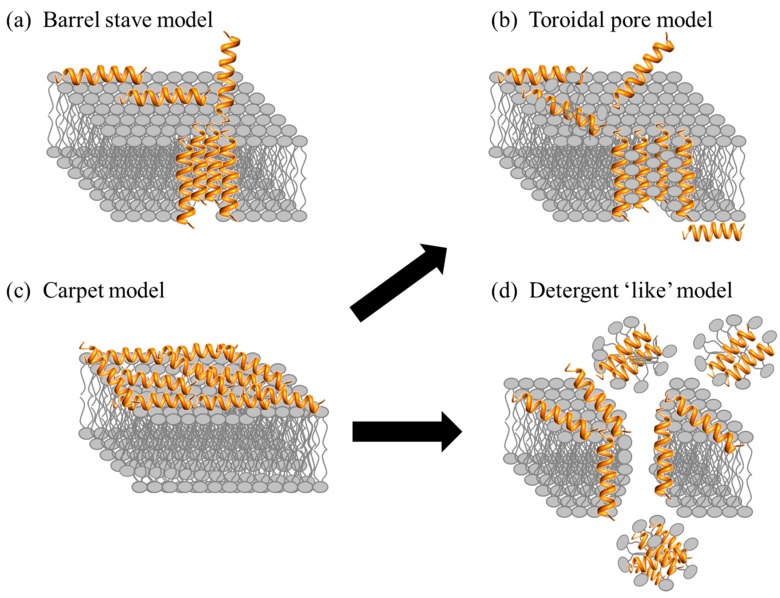
Proposed mechanisms of action for AMPs in bacteria. See text for description of the various mechanisms and examples.

After the initial electrostatic and hydrophobic interactions, the AMPs accumulate at the surface and self-assemble on the bacterial membrane after reaching a certain concentration [95,96]. At this stage various models have been used to describe the action of AMPs. The models can be classified under two broad categories: transmembrane pore and non-pore models. The transmembrane pore models can be further subdivided into the barrel-stave pore and toroidal pore models. In the barrel stave model, the AMPs are initially oriented parallel to the membrane but eventually insert perpendicularly in the lipid bilayer [97] (Figure 5a). This promotes lateral peptide-peptide interactions, in a manner similar to that of membrane protein ion channels. Peptide amphipathic structure (α and/or β sheet) is essential in this pore formation mechanism as the hydrophobic regions interact with the membrane lipids and hydrophilic residues form the lumen of the channels [30,98]. A unique property associated with AMPs in this category is a minimum length of ~22 residues (α helical) or ~8 residues (β sheet) to span the lipid bilayer. Only a few AMPs, such as alamethicin [99], pardaxin [100,101] and protegrins [30], have been shown to form barrel stave channels.

Furthermore, in the toroidal pore model, the peptides also insert perpendicularly in the lipid bilayer but specific peptide-peptide interactions are not present [99]. Instead the peptides induce a local curvature of the lipid bilayer with the pores partly formed by peptides and partly by the phospholipid head group (Figure 5b). The dynamic and transient lipid-peptide supramolecule is known as the “toroidal pore”. The distinguishing feature of this model as compared to the barrel-stave pore is the net arrangement of the bilayer: in the barrel-stave pore, the hydrophobic and hydrophilic arrangement of the lipids is maintained, whereas in toroidal pores the hydrophobic and hydrophilic arrangement of the bilayer is disrupted. This provides alternate surfaces for the lipid tail and the lipid head group to interact with. As the pores are transient upon disintegration, some peptides translocate to the inner cytoplasmic leaflet entering the cytoplasm and potentially targeting intracellular components [102]. Other features of the toroidal pore include ion selectivity and discrete size [69]. A number of AMPs such as magainin 2 [45], lacticin Q [45], aurein 2.2 [39] and melittin [45,99] have been shown to form toroidal pores. In addition, the type of pore formed by aurein 2.2 has been shown to depend on the lipid composition: in a 1-palmitoyl-2-oleoyl-*sn*-glycerol-3-phosphocholine (POPC)/1-palmitoyl-2-oleoyl-*sn*-glycero-3-phospho-(1′-*rac*-glycerol) POPG (1:1) membrane model, the peptides induce toroidal pores, whereas in a 1,2-dimyristoyl-*sn*-glycero-3-phosphocholine (DMPC)/1,2-dimyristoyl-*sn*-glycero-3-phospho-(1′-*rac*-glycerol) DMPG (1:1) membrane model, the peptides work in a detergent-like model (details below) indicating the importance of the hydrophobic thickness of the lipid bilayer and the membrane composition [103,104]. Ultimately, both pore forming models (toroidal pore and barrel) lead to membrane depolarization and eventually cell death.

AMPs can also act without forming specific pores in the membrane. One of these models is designated as the carpet model [45,69,82]. In this case, the AMPs adsorb parallel to the lipid bilayer and reach a threshold concentration to cover the surface of the membrane, thereby forming a “carpet” (Figure 5c). This leads to unfavorable interactions on the membrane surface. Consequently, the membrane integrity is lost, producing a detergent-like effect, which eventually disintegrates the membrane by forming micelles. The final collapse of the membrane bilayer structure into micelles is also known as the detergent-like model (Figure 5d). The carpet model does not require specific peptide-peptide interactions of the membrane-bound peptide monomers; it also does not require the peptide to insert into the hydrophobic core to form transmembrane channels or specific peptide structures [69]. Many peptides act as antimicrobial agents despite their specific amino acid composition or the length of the sequence. Such AMPs typically act using the carpet model [82], and do so at high concentrations because of their amphiphilic nature [2]. Examples of AMPs acting by the carpet model are cecropin [105], indolicidin [62], aurein 1.2 [106], and LL-37 [82].

Overall, there are a number of models to describe the MOA of AMPs. In addition to those given above, there are other related models such the Shai-Huang-Matsazuki model, the interfacial activity model, and the electroporation model [45]. Some models do not make the specific distinctions shown in Figure 5. For example, it has been suggested that the carpet-like mechanism is a prerequisite step for the toroidal pore model [45]. Most studies to elucidate the MOA of AMPs involve the use of model membranes. The mode of action of only a few AMPs has been investigated with whole bacterial cells using imaging techniques [107,108]. It is possible that different results may be obtained using different membrane models or assay conditions, for example more than one MOA is possible for certain AMPs such as BP100 as the peptide-to-lipid ratio changes [109] indicating that the models described here may or may not translate directly to what is occurring in bacteria.

### 2.2. Direct Killing: Non Membrane Targeting Mechanisms of Action

**Figure 6:**
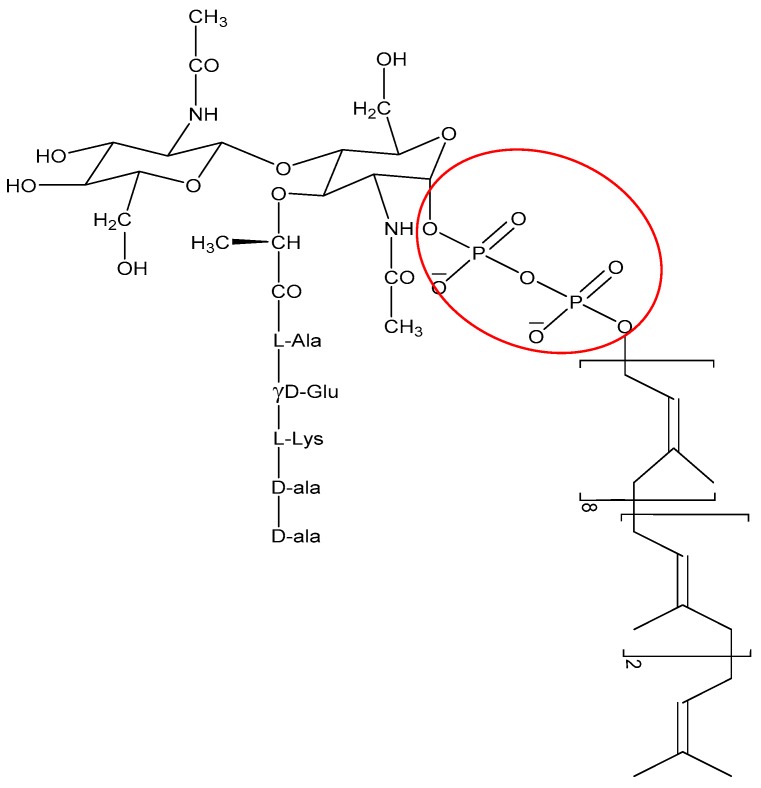
Structure of lipid II molecules with the pyrophosphate moiety circled in red.

The non-membrane targeting AMPs can be divided into two broad categories: those that target the bacterial cell wall and those that have intracellular targets (Figure 3). Similarly to conventional antibiotics such as penicillin, AMPs can also inhibit cell wall synthesis. Although most conventional antibiotics bind to specific proteins involved in the synthesis of the cell wall components, AMPs often interact with various precursor molecules that are required for cell wall synthesis. One molecule that is a prime target is the highly conserved lipid II [110]. For instance, AMPs such as defensins bind to the negatively charged pyrophosphate sugar moiety of the lipid II molecule [111] (Figure 6). The binding event can further promote formation of pores and membrane disruption [110]. AMPs such as the human β defensin 3 [111] and α defensin 1 [112] rely on selective binding to lipid II to confer bactericidal activity.

When AMPs were first discovered, it was thought that they could not have intracellular targets. Studies with the original α helical peptides such as magainin, cecropin and melittin showed that an all-d version of these peptides was equipotent compared to the natural all-l peptides [113], supporting the idea that stereospecific targets such as proteins or DNA/RNA were not required for antibacterial activity, further confirming that AMPs target the membrane [88]. However, subsequent studies revealed that other AMPs with all-d or all-l amino acids did not have equal activity [114]. Now it is well established that several AMPs have intracellular targets as the AMPs do not cause membrane permeabilization at the minimal effective concentration, but still cause bacterial death [30].

Mechanistically, these AMPs interact with the cytoplasmic membrane first and then accumulate intracellularly, where they can block critical cellular processes. Many novel mechanisms involving intracellular targets, such as inhibition of protein/nucleic acid synthesis and disruption of enzymatic/protein activity, have been discovered [30]. For example, buforin II, an histone derived AMP from frogs, translocates through the bacterial membrane without permeabilization and binds to the DNA and RNA of *E.coli* [52]. Human α defensin 5 also translocates into the cytoplasm of *E.coli* and accumulates at the cell division plate and at opposite poles suggesting part of the antibacterial activity might be due to the targets in the cytoplasm. Indolicidin [115], human β defensin 4 [116], human α defensin 1 [55] and PR-39 [117] have also been shown to target intracellular bacterial components.

### 2.3. Immune Modulation Mechanism of Action 

In addition to the direct killing of microbes, AMPs can also recruit and activate immune cells (Figure 3), resulting in enhanced microbial killing and/or control of inflammation [21,118,119]. As AMPs are produced by many immune cells such as neutrophils and macrophages, they are one of the first molecules that encounter invading microbes [2]. In an infection, it is important to produce an immune response to attract other immune cells and also control inflammation. Interestingly, some AMPs can produce a variety of immune responses: activation, attraction, and differentiation of white blood cells; stimulation of angiogenesis; reduction of inflammation by lowering the expression of proinflammatory chemokines; and controlling the expression of chemokines and reactive oxygen/nitrogen species [21,25,118,120,121].

The human AMPs such LL-37 and β defensins have the ability to attract (chemoattract) immune cells such as mast cells [122], leukocytes [123] and dentritic cells [124]. Innate defense regulators (IDR) which are synthetic versions of natural AMPs, such IDR-1 and IDR-1018, also suppress pro-inflammatory cytokines in mice infection models [125,126]. IDR-1018 has also shown promise in reducing the inflammation response in severe malaria, without having direct anti-malaria activity. Mice treated with a combination of anti-malaria agents and IDR-1018 demonstrated a reduction in the harmful neural inflammation which otherwise leads to death. Although most AMPs have been shown to interact with innate immune system components such as neutrophils and macrophages, there is evidence that they are also involved in modulation of the adaptive immune system, i.e., the T and B cells. The exact mechanisms are not well understood [21], however, some studies show that AMPs may act as vaccine adjuvants [121,127]. Interestingly, all these studies show that AMPs work in many independent or co-operative “multi-hit” [128] mechanisms of action, making AMPs ideal candidates for future development.

## 3. Challenges with Antimicrobial Peptides

Although many eukaryotic AMPs have been identified and characterized, not many have made it to clinical trials (Table 2) and only a few have been approved by the US Food and Drug Administration (FDA). Most AMPs in clinical trials are analogues of natural AMPs, but there are some that are completely synthetic (e.g., IMX942). The majority of AMPs in clinical trials are limited to topical applications, due to systemic toxicity, susceptibility of the peptides to protease degradation and rapid kidney clearance [2,96,129] of these peptides if they are ingested. Oral administration of AMPs can lead to proteolytic digestion by enzymes in the digestive tract such as trypsin and pepsin. Moreover, systemic administration results in short half lives in vivo, protease degradation and cytotoxic profiles in blood [22]. Many strategies have been investigated to circumvent these issues and to improve the efficacy of AMPs. These include chemical modification of AMPs [130] and the use of delivery vehicles [131]. These strategies will be discussed in great detail below.

## 4. Strategies to Improve Antimicrobial Peptides

### 4.1. Chemical Modification of AMPs

Various chemical modifications of AMPs have been utilized to improve the stability of peptides against proteolytic digestion including the use of d-amino acids, cyclization, acetylation and peptidomimetics. Incorporation of non-natural d-amino acids into AMP sequences reverses the stereochemistry of the peptide and hence prevents protease degradation, as enzymes are stereospecific. 

**Figure 7:**
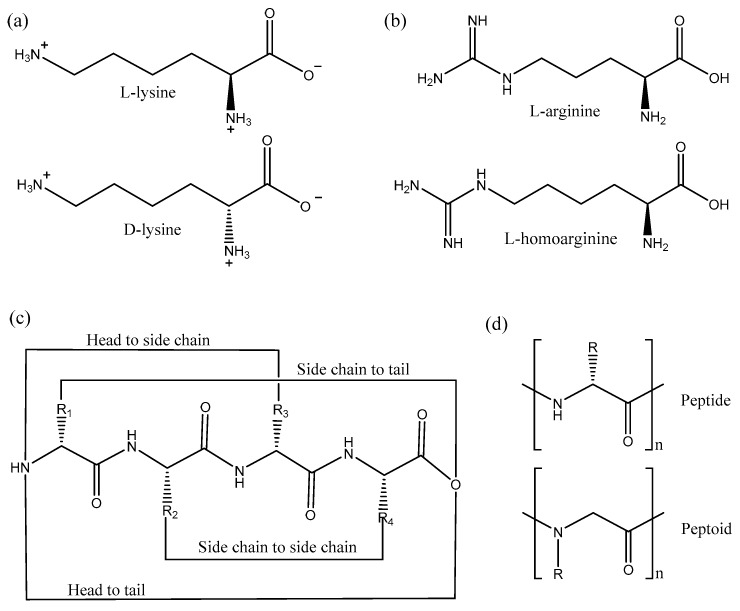
Chemical modifications used to improve AMP properties: (**a**) use of d-amino acids such as lysine, (**b**) use of non-natural amino acids such as l-homoarginine, (**c**) various cyclization strategies, (**d**) use of a peptoid (1-dimensional structural difference relative to a peptide shown).

In a recent study, Zhao et al. isolated a lysine rich AMP from the venom of the social wasp (*Polybia paulista*), MPI, which was active against Gram-positive and Gram-negative bacteria and also fungi [132]. In order to prevent proteolytic digestion by trypsin the authors designed two peptides, one with all the amino acids replaced with d-amino acids, d-MPI, and the other peptide with only the lysine residues substituted with its d-amino acid (Figure 7a), d-lys-MPI, as trypsin cleaves after positively charged amino acids such as lysine [132]. Interestingly, both the peptides, d-MPI and d-lys-MPI were resistant to trypsin digestion, however only d-MPI was equipotent in terms of activity when compared to MPI. d-lys-MPI was inactive because the secondary structure was destabilized upon introduction of the d-amino acids. d-MPI adopted a right-handed α helical conformation, whereas the d-lys-MPI did not adopt any regular structure. In a similar study, d-BMAP28, a peptide from bovine myeloid, was made proteolytically stable by replacing all amino acids by the d-counterparts. d-BMAP28 remained equipotent in terms of both its antimicrobial and immunomodulatory activities when compared to BMAP38 [133]. Moreover, Falciani et al. reported that another AMP d-M33 was more active against biofilms formed by Gram-positive bacteria, as compared to M33 [134]. Overall, the use of d-amino acids in AMPs leads to retention of the antimicrobial activity, while preventing proteolysis. This confirms that these AMPs interact with the bacterial membrane without making use of specific receptors [22], since the stereochemistry of the amino acids has no impact on membrane binding. Finally, it should be emphasized that the synthesis of peptides containing d-amino acids is very costly [135]. Alternative strategies are thus important to reduce the economic impact.

These alternative approaches are many and varied. For instance, the substitution of positively-charged arginine in a sequence with other charged non-natural amino acids, such as l-ornithine and l-homoarginine (Figure 7b), also increases proteolysis stability of AMPs [137]. Moreover, acetylation of the N-terminus also increases the proteolytic stability of peptides as it blocks the activity of aminopeptidases; however, this leads to removal of a positive charge which in most cases decreases the antimicrobial activity [138,139]. Cyclization of peptides by different methods also prevents protease degradation (Figure 7c). Cyclization by joining the backbone N- and C-termini or by disulfide bridges similar to human defensins are common strategies used to increase serum stability of AMPs [140]. In a recent study, click chemistry was developed for specific cyclization of certain amino acids [141]. The results suggested that the α helical structure was critical for activity as the i, i + 4 cyclization (1st and 4th amino acid cyclized) retained the structure and activity compared to i, i + 6 cyclization, which was not structured and inactive. 

Further strategies include the use of peptidomimetics: peptide-like polymers made from a backbone that is altered when compared to a peptide [142,143,144]. The main concept in the use of peptidomimetics is to maintain activity by conserving the 2D and 3D spatial arrangement of the side chains, but modify the backbone to prevent proteolysis. Some examples of peptidomimetics include peptoids, ceragenins, oligoacyllysines and β-peptides [143,144]. Peptoids are isomers of peptides in which the side chain is bonded to the backbone nitrogen instead of the alpha carbon making them resistant to protease degradation [145] (Figure 7d). Peptoids derived from pexiganan have been shown not only to mimic the 1D structure but also mimic the 2D structure, function and mechanism of action of pexiganan [146]. CD studies confirmed that peptoids adopt α helical structure in the presence of phospholipids, whereas X-ray reflectivity showed peptoids bind to the membrane and are membrane active [146]. Cyclization of peptoids also enhances the membrane permeation properties leading to better antimicrobials [147].

### 4.2. Delivery Systems for AMPs

Another important strategy to improve the properties of AMPs is to use delivery systems, i.e., systems ranging from inorganic and polymer materials, surfactant/lipid self-assembly systems to peptide self-assembly systems [131,148] which can be used to improve the stability, toxicity, half-life and release profile of AMPs. The AMPs can be covalently attached to the delivery system or non-covalently encapsulated by these different types of systems. Below we will briefly mention the different kinds of delivery systems, but then mainly focus on polymer conjugates.

Many inorganic materials such as mesoporous silica particles [149], titanium [150], metal nanoparticles (mostly Au [151] and Ag [152]), quantum dots [153], graphene [154] and carbon nanotubes [155] have been utilized for AMP delivery systems. Mesoporous nanoparticles can be obtained from various materials, but silica nanoparticles have been used extensively due to their ability to form well defined pores for the loading of AMPs [156]. Other inorganic materials e.g., graphene oxide nanotubes can be used to covalently attach AMPs such as nisin to enhance the antimicrobial activity against methicillin resistant *Staphylococcus aureus* (MRSA) [154]. For a comprehensive review of delivery systems, the reader is referred to Nordström et al. [131] and Urbán et al. [157].

Lipids and surfactants can form a wide range of structures such as micelles, liposomes and microemulsions [158]. From their mechanism of action, it is well known that AMPs interact with lipids/surfactants and hence lipid/surfactant-based delivery systems of peptides are well studied [159]. The encapsulation of LL-37 within liposomes composed of 1,2-distearoyl-*sn*-glycero-3-phosphocholine (DSPC)/1,2-distearoyl-*sn*-glycero-3-phospho-ethanola-mine-*N*-[amino(polyethylene glycol)] (DSPE-PEG)/cholesterol ensured enhanced bioactivity and reduced toxicity towards cell cultures [160]. In a similar study, DSPC/1,2-distearoyl-*sn*-glycero-3-phospho-(1′-*rac*-glycerol) (DSPG) liposomes were found to encapsulate nisin and protect it from extreme alkaline/acidic conditions and elevated temperature [161]. Moreover, antimicrobial emulsifiers such as monolaurin can be used to load AMPs such as nisin Z to form nano-micellar structures that display synergistic antimicrobial activity against *Staphylococcus aureus* [162].

Polymeric materials can also be utilized in various ways for the delivery of AMPs. These include polymer particles and fibers, polymer gels, polymer multilayers and polymer conjugates [131]. In a recent study, colistin-loaded poly(lactic-co-glycolic acid) (PLGA) particles coated with chitosan were used to increase transport efficiency through cystic fibrosis mucus [163]. On the other hand, Yüksel et al. employed magainin 2 immobilized PLGA electrospun fibers loaded with epithelial growth factors for better wound healing and lowering bacterial adhesion [164]. Hydrogels made from amphiphilic poly(2-(adenine-9-yl) ethanol methacrylate-co-sulfobetaine methacrylate) polymer can also be utilized to load AMPs such as alamethicin. This assembly complex displayed better antimicrobial activity compared to free peptide due to protection against protease degradation [165].

**Figure 8:**
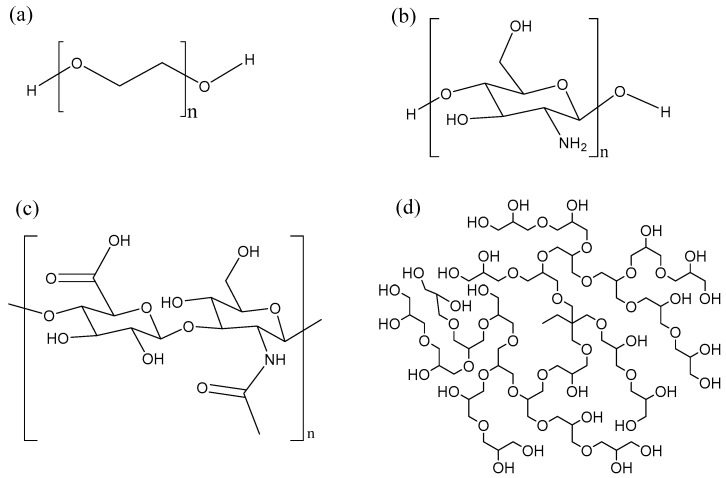
Various polymers used for AMP conjugation: (**a**) polyethylene glycol (PEG), (**b**) chitosan, (**c**) hyaluronic acid, (**d**) hyperbranched polyglycerol (HPG).

Polymer conjugation was one of the first techniques used to enhance the properties of biomolecules such as proteins, peptides and nucleic acids [166]. PEGylation is a process by which a polyethylene glycol chain (Figure 8a) is added to a biomolecule. PEGylation is one of the most extensively investigated strategies to improve the performance of proteins, peptides and other biomolecules. The advantages of PEGylation include reduced non-specific uptake in tissue, reduced cell toxicity, increased blood half-life and reduced proteolytic degradation [148,167]. Many AMPs such as tachyplesin I [168], magainin 2 [169], nisin [170] have been PEGylated, leading to improved properties. However, this improvement is often at the expense of the antimicrobial activity. For instance, PEGylation of the cyclic peptide tachyplesin and of magainin reduced toxicity towards CHO-K1 cells, but also decreased antimicrobial activity towards *E. coli* and *S. epidermidis*. Interestingly, PEGylation of nisin via the amine group of the lysine side-chain lead to a conjugate that was inactive. It was hypothesized that the amino group of the lysine residue is needed to bind to the pyrophosphate of the lipid II molecule, hence the loss of activity. In other words, the site and nature of the conjugation chemistry [170] has an impact on the properties of the resulting compounds. Finally, PEGylation of KYE28 revealed that increasing the length of PEG lead to a partial loss in antimicrobial activity, but also to a strong decrease in hemolysis and to improved selectivity in blood and bacteria mixtures [171]. Important drawbacks of linear PEG is its relatively large hydrodynamic size, lack of multiple functionality and high intrinsic viscosity in aqueous conditions which increases with increase in molecular weight [172].

Alternatively, AMPs can also be conjugated to biopolymers such as chitosan and hyaluronic acid, which have multiple functional groups for the attachment of peptide. Chitosan (Figure 8b) is a linear biocompatible, biodegradable carbohydrate polymer with some antimicrobial activity [173]. Conjugation of the short and moderately active anolin to chitosan increased the antimicrobial activity of the conjugate and abolished the hemolytic activity [174]. In general, the antimicrobial activity increased in proportion to the number of peptides attached to the chitosan polymer. Recently, click chemistry has been used as an interesting approach to attach AMPs to chitosan at specific sites [175].

Another biocompatible, biodegradable linear carbohydrate polymer utilized for AMP conjugation is hyaluronic acid (Figure 8c). In contrast to PEGylation, conjugation of nisin to hyaluronic acid results in a conjugate which maintains antimicrobial activity [176]. The hyaluronic-nisin conjugates were mostly active against Gram-positive bacteria, but in the presence of ethylenediaminetetraacetic acid (EDTA) the antimicrobial activity extended towards Gram-negative bacteria. The EDTA binds to divalent cations such as Mg^2+^ and disturbs the lipopolysaccharide-divalent cation interaction, disintegrating the outer membrane of the Gram-negative bacteria, making it more susceptible to the hyaluronic-nisin conjugate.

Moreover, we have shown that hyperbranched polyglycerol (HPG) (Figure 8d) is as or even more biocompatible than PEG [177,178,179]. HPG is a hyperbranched polymer with many hydroxyl groups available for modification. HPG has been utilized for the development of long circulating drug conjugates [178,180], anticoagulant neutralizing agents [177,181], for cell surface modification [182], as an osmotic agent in peritoneal dialysis [183] and cell preservation [184], making it an excellent candidate to conjugate AMPs. In our initial studies, we conjugated antimicrobial peptide aurein 2.2 to 44 kDa HPG and were able to obtain a conjugate with better therapeutic index [185]. The HPG-aurein conjugates were not toxic to cell cultures and red blood cells at twice the new minimum inhibitory concentration (MIC) and had excellent blood properties compared to the aurein peptide only. In a more recent study, we were able to develop analogues of aurein peptides with better antimicrobial activities [186]. These new peptides were conjugated to HPG of different molecular weights and it was found that the antimicrobial activity of the peptides increased with a decrease in molecular weight of HPG even though the peptide density was kept constant. As expected, the blood and cell culture properties of the conjugates were improved compared to the peptides alone with the 22 kDa HPG-peptide conjugate having the best therapeutic index. Interestingly, upon treatment with trypsin, the 22 kDa HPG-peptide conjugates were resistant to proteolytic digestion and retained antimicrobial activity whereas the free peptide degraded and was inactive. Conjugation of AMPs to HPG could be a general strategy used for desirable therapeutic properties.

## 5. Conclusions

Antimicrobial peptides are unique molecules that are promising candidates to treat multidrug-resistant organisms. Interestingly, AMPs have many mechanisms of action and can also display antiviral and anticancer activities. Over the last decade, different strategies have been utilized to improve the efficacy of AMPs to push forward the development as therapeutic agents. This review examined various approaches from modifying current and designing new AMPs to using delivery systems for AMPs. Chemical modification of AMPs is one of the most frequent and easiest methodologies employed to improve activity and biocompatibility. In recent years many covalent and non-covalent delivery systems have been developed to further the pursuit. Overall, due to the emergence of antibiotic resistance and the fact that many AMP-based drugs are in clinical trials or under development, the next decade will reveal the benefits of these novel compounds and lead to commercialization.

## Figures and Tables

**Table 1:** Classes of antimicrobial peptides based on structure.

Category ^a^	Peptides	Unique Structural/Sequence Feature	Source
α helical peptides	Aurein 1–2 [36,37]	Amidated C-terminus	Frogs
	Mellitin [23]	Amidated C-terminus	Bees
	Brevinin 1 [48]	-	Frogs
	Maculatins [50]	Amidated C-terminus	Frogs
	Citropin [51]	Amidated C-terminus	Frogs
	Buforin II [52]	-	Toad
	Cathelicidins [31]		
	• LL-37 ^b^	Amidated C-terminus	Humans
	• BMAP-27,28,34 ^b^	-	Bovine
	• Magainins	-	Frogs
	• Cecropin	Amidated C-terminus	Insect
β sheet peptides	Cathelicidins [31]		
	• Protegrins	Cysteine rich	Pigs
	• Bactenecin	Disulfide forming loop/Arginine rich	Bovine
	Defensins ^c^ [20,53,54,55]		
	• α defensins	Three disulfide bonds	Mammals
	• β defensins	Three disulfide bonds	Mammals
	• θ defensins	Three disulfide bonds and cyclic	Gorilla
	Tachyplesins [16] and Polyphemusin [49]	Cysteine/arginine rich and amidated C-terminus	Horse Crab
Extended/flexible	Cathelicidins [31]		
	• PR-39 ^b^	Proline and arginine rich	Pigs
	• Tritrpticin	Tryptophan and arginine rich	Pigs
	• Indolicidin	Tryptophan and amidated C-terminus	Bovine
	• Crotalicidin 15–34	Lysine rich	Snakes
	Histatins [56]	Histidine rich and amidated C-terminus	Humans

^a^ Classification is based on the predominant structure. Some peptides might have mixed α helix and β sheet. ^b^ LL-37 is also known as cathelicidin antimicrobial peptide, 18 kDa or CAP-18; BMAP stands for bovine myeloid antimicrobial peptide; and PR stands for proline-rich cathelicidins. ^c^ Analogues of defensins are also found in insects, plants and fungi and are not restricted to mammals.

**Table 2:** Antimicrobial peptide in clinical trials [22,35,118,136].

Peptide	Progress	Application	AMP Analogue (Host)
Pexiganan	Phase III	Topical application for diabetic foot ulcers	Magainin (frogs)
OP145	Phase I/II	Bacterial ear infection	LL-37 (humans)
Omiganan	Phase III	Topical cream for prevention of catheter infection, severe acne, rosacea, atopic dermatitis	Indolicidin (bovine)
PAC 113	Phase II	Mouth wash for fungal/yeast infection	Histatin (humans)
Iseganan	Phase III	Treatment of inflammation and ulceration of digestive system mucous membrane	Protegrin-1 (pigs)
IMX942	Phase II	Intravenous administration against hospital-acquired bacterial infections	Synthetic analogue of IDR-1 ^a^

^a^ IDR stands for innate defense regulator.
